# 3D-Printed Titanium Custom-Made Prostheses in Reconstruction after Pelvic Tumor Resection: Indications and Results in a Series of 14 Patients at 42 Months of Average Follow-Up

**DOI:** 10.3390/jcm10163539

**Published:** 2021-08-12

**Authors:** Carmine Zoccali, Jacopo Baldi, Dario Attala, Alessandra Scotto di Uccio, Luca Cannavò, Gennaro Scotto, Alessandro Luzzati

**Affiliations:** 1 Oncological Orthopaedics Department, IRCCS—Regina Elena National Cancer Institute, Via Elio Chianesi 53, 00144 Rome, Italy; jacopo.baldi@ifo.gov.it (J.B.); dario.attala@ifo.gov.it (D.A.); 2 Hepato-Biliary and Organ Transplant Unit, School of General Surgery, Sapienza University, Viale del Policlinico 155, 00161 Rome, Italy; allascotto@gmail.com; 3 Oncological and Reconstructive Surgery Unit, IRCCS—Galeazzi Orthopedic Institute, Via Riccardo Galeazzi 4, 20161 Milan, Italy; lucacannav@hotmail.it (L.C.); g.scotto1@virgilio.it (G.S.); alessandroluzzati@gmail.com (A.L.)

**Keywords:** pelvic tumors, custom-made prosthesis, wide resection, pelvic reconstruction, additive manufacturing

## Abstract

Wide resection is currently considered the mainstay treatment for primary bone tumors. When the tumor is located in anatomically complex segments, 3D-Printed Titanium Custom-Made Prostheses (3DPTCMP) are possible reconstructive solutions. The aim of the present paper is to analyze indications, results and complications of a series of 14 patients who underwent pelvis reconstruction with 3DPTCMP after tumor removal from January 2015 to December 2019. Chondrosarcoma was the main histology; indications were tumors located in the acetabular area without enough residual bone to support a cup with an iliac stem, and tumors located near the sacrum-iliac joint. The margins were wide in 12 cases, and marginal and intralesional in one case each. In three cases, resection also included the sacrum-iliac joint, so a spine stabilization was performed and linked to the pelvic prosthesis; The average MSTS score was 46.3%; the 5-year local recurrence-free survival was 85.7%. Wound dehiscences were the main complication, resolved with multiple debridements; nevertheless, prosthesis removal was necessary in one case. Currently, the 3DPTCMP is an effective resource for reconstruction after resection of tumors located in the pelvis. Further studies are necessary to value long-term results; more strategies are necessary to try to reduce the infection rate and improve osteointegration.

## 1. Introduction

Wide resection is currently considered the mainstay treatment for primary bone tumors [1,2,3]; it is also suitable for metastatic patients with long survival, obviously taking the invasiveness of the tumor into account [4,5].

When the tumor is located in limbs, reconstruction after resection is usually performed with modular prostheses which are able to reconstruct the joint and restore limb length [6,7].

When the tumor is located in anatomically complex segments, such as in the pelvis or the scapula, no efficient modular prostheses are commercially available.

Several reconstruction methods have been proposed: massive autograft, which is still considered the gold standard, autoclaved autograft, arthrodesis or pseudoarthrosis [8,9,10,11]. However, these possibilities present several complications and poor results, so custom-made implants, that potentially have better results, must be considered [12].

In the past, the technology used to produce a custom implant was based on subtraction techniques. This meant high manufacturing costs, long production times and small distribution, limited to just some centers.

The development of additive technologies using titanium powders and their application in prosthesis production is broadening the use of these techniques, and further in reconstruction after pelvis tumor removal, where reconstruction is not efficient using standard modular prostheses and allografts or composite prostheses.

The aim of the present study is to analyze indications, techniques and complications of a series of 14 patients who underwent pelvis reconstruction with 3D-Printed Titanium Custom-Made Prosthesis (3DPTCMP) after tumor removal, presenting some hot topics.

Particular emphasis was reserved for the analysis of still-unresolved problems.

## 2. Materials and Methods

All patients who underwent pelvic resection for primary tumors and reconstruction with 3DPTCMP in two tertiary research hospitals from January 2015 to December 2019 were reviewed.

Fourteen patients, 8 females and 6 males, with an average age of 51.1 years (range 15–74 years, median 55 years) were enrolled in the study (Table 1).

All cases were discussed in a multidisciplinary team meeting to assure the best possible treatment.

Diagnoses were performed on the histologies, reviewed by two expert pathologists, one per center, trained in muscular skeletal tumors. 

The tumor site was described using the Dunham and Enneking classification which identifies four areas of involvement: type 1 corresponds to the iliac wing, type 2 to the acetabular area, type 3 to the ischium and the two ileo-ischium-pubic rami and type 4 to the sacrum and the sacrum-iliac joint [13].

Tumor extension was also evaluated using the Enneking staging system for malignant musculoskeletal tumors, based on surgical grade, local extent and presence or absence of metastases [14].

A total-body CT scan was carried out to exclude distant metastases; a high-resolution pelvic CT-scan with 1 mm slices was performed for prostheses production, whereas a gadolinium-enhanced pelvis MRI was conducted to better identify disease limits and establish cutting margins.

Dicom files were sent to the company in order to produce a 3D-computerized reconstruction of the pelvis and the tumor. Then, the surgical team established the osteotomies based on the MRI and the company engineers designed the prosthesis to fill the gap.

All 3DPTCMPs were produced by the same company (MT-ORTHO srl, Aci Sant’Antonio (CT), Italy) on the specific requests and with the characteristics required by the two surgeons.

The prostheses were composed of two different parts: the body, made of solid titanium, and the bone-prosthesis interfaces, made of a porous titanium structure to enhance bone ingrowth. 

The company also provided specific cutting guides to perform the osteotomies established during 3DPTCMP designing. Spine hardware and hip prostheses were chosen on the basis of surgeon preferences and hospital availability.

The cups were cemented in the prosthetic acetabulum using acrylic cement [15].

The average cost for a pelvic prosthesis was about 12,000 EUR (10,000 EUR–14,000 EUR); the specific price depended on the complexity of the project, the difficulty of production and the size of the prosthesis.

The cost of the hip prosthesis (from 1500 EUR for a standard hip prosthesis to 6000 EUR for a megaprosthesis) and the spine stabilization system (3000–6000 EUR based on its length) have to be added to the cost of the pelvic prosthesis. 

Surgeries were performed by two surgeons, one per center, both trained in pelvic and spinal oncologic surgery. All surgeries were performed in one stage, except for two in which spine stabilization was performed before or after pelvic tumor resection.

Meropenem (1 g for three administrations per day, MERREM, Pfizer, New York, NY, USA) and Vancomycin (1 g for two administrations per day, Vancomicina Pfizer, New York, NY, USA) were administered as antibiotic prophylaxis and usually maintained for 10 days.

Complications are distinguished as early or late if they occurred before or after 30 days from surgery, respectively [16]. Blood loss was defined as massive when it exceeded the circulating blood volume in 24 h or 50% of circulating blood volume in 3 h. Hypotension was diagnosed when mean systemic pressure was lower than 85 mmHg for more than 15 min. Urological problems included urinary retention and urinary incontinence or ureteral damage.

The postoperative rehabilitative protocol was discussed case by case, considering the patient’s and the surgery’s specific characteristics. 

The MSTS score was used to value functional results and patient satisfaction [17].

All patients performed post-operative X-rays and CT scan to check the correct positioning of the prostheses and screws; thereafter, X-rays were repeated at 40 days and every three months for the first two years, every four months during the third year and every six months during the fourth and the fifth years for high-grade malignancies; every four months for the first two years and every six months until the fifth year from index surgery for low-grade malignancies.

General checks were established based on the specific diagnosis.

Recurrences and survival were valued considering 5-year Local Recurrence-Free Survival (5y-LRFS), 5-year Distant Metastasis-Free Survival (5y-DMFS), and 5-year Overall Survival (5y-OS). The 5-year disease-specific survival was not considered a good estimator because three out of four deaths, although not directly caused by the tumors, were probably indirect consequences of the surgeries.

The related Kaplan–Meier curves are reported.

Statistical Analysis: a descriptive statistic was used to analyze the data. All analyses were performed using Microsoft Excel (Redmond, Washington, USA) and Stat Plus (AnalystSoft Inc., Walnut, CA, USA).

The study was conducted in accordance with the Declaration of Helsinki Ethical Principles and Good Clinical Practices and was approved by the IRCCS Lazio-IFO Section Central Ethic Committee (Reg. Sper. N.1380/20).

All patients provided their consent to the use of their data for research purposes and publication.

## 3. Results

The patients’ main characteristics and results are reported in Table 1 and summarized in Table 2.

### 3.1. Epidemiology

The present series was composed of 14 patients, six males and eight females, with an average age of 52.1 years (range 15–74 years, median 55 years) (Table 1). 

### 3.2. Histologies and Localizations

Diagnosis was performed on the specimens obtained from CT-guided trocar biopsies in 11 cases, from ultrasound-guided trocar biopsy in one case, from open minimally invasive biopsy in one case, and from a previous intralesional surgery performed in a non-specialized center in another case.

The histologies were Chondrosarcoma in 11 cases, whereof one was mesenchymal Chondrosarcoma and in five, four and one cases were G3, G2 and peripheral G1 Chondrosarcomas, respectively; the remaining cases were two Ewing sarcomas and one metastatic epithelioid hemangioendothelioma. 

Following the Enneking and Dunham classification, nine cases involved the T2–T3 areas (acetabulum and pubic rami), three cases the sacrum-iliac joint, whereof one was in T1–T2–T4 areas and two in T1–T4 areas. Considering Enneking’s staging, four cases were stage IB, two cases stage IIA, seven cases stage IIB and one case stage III.

### 3.3. Symptoms

**Figure 1:**
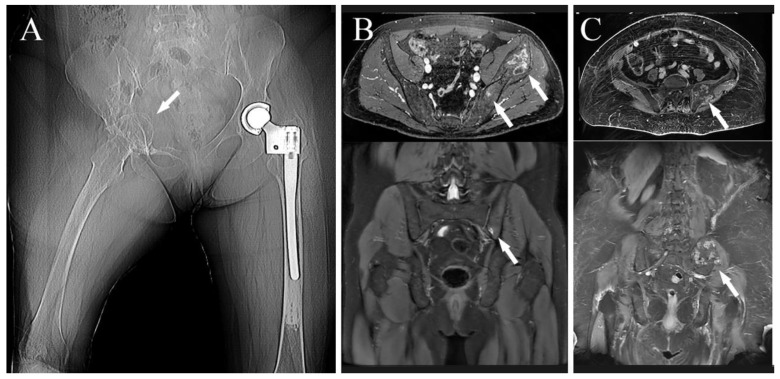
(**A**) Preoperative X-ray showing a right hip fixed in flection in a patient affected by metastatic hemangioendothelioma (white arrow), two years before she underwent left proximal femur replacement for pathological fracture (case 6); (**B**) preoperative MRI of a patient affected by Ewing’s sarcoma of the left hip involving the sacrum-iliac joint as well (white arrows) (case 7); (**C**) preoperative MRI showing a patient affected by a G2-chondrosarcoma of the left sacrum-iliac joint (white arrows) (case 2).

Localized pain was present in 13 out of 14 cases; in the last case, the disease was accidently discovered because of an MRI performed for low-back pain with sciatic irradiation due to disk degeneration (case 8); neurological symptoms, related to the diseases, were present in five cases, sciatic and crural irradiation in three and one cases, respectively; neurological bladder was already present at diagnosis in the last case (case 11); one patient presented flexed anchylosis of the right hip due to a pathological proximal femur fracture (Figure 1A) (case 6).

### 3.4. Surgeries

**Figure 2:**
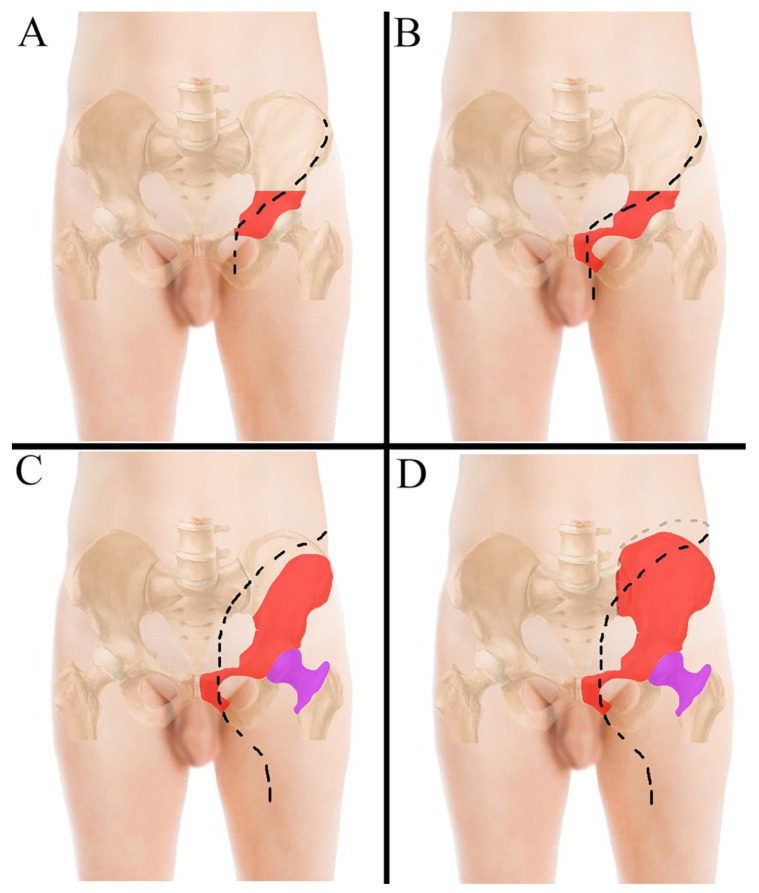
(**A**) ileo-inguinal incision performed to resect tumors located in the acetabular area; (**B**) ileo-inguinal incision performed to resect tumors located in the acetabulum with symphysis involvement; in this case the incision bends slightly proximally to cross the inguinal ligament more medially. (**C**) incision performed to resect tumors located in the iliac wing, the hip and the symphysis; (**D**) incision performed when the tumor also involves the sacrum-iliac joint. The tumor areas in the pelvis and in the femur are drawn in red and magenta, respectively.

No preoperative angiographies and embolizations were performed. The tumor involved the acetabulum in 12 cases, of which one also involved the sacrum-iliac joint (Figure 1B) (case 7); in the last two cases the tumor involved the sacrum-iliac joint, sparing the acetabulum (Figure 1C) (case 1, 14). When the tumor involved the acetabulum (Figure 2A), the patient was positioned in the supine or lateral position (the latter is preferred if the tumor extends to the iliac wing) and an ileo-inguinal incision was performed (Figure 2B), prolonged medially if the symphysis was also involved and then prolonged distally along the thigh. 

The ileo-inguinal approach is the basis for resection of pelvic-bone tumors and reconstruction. 

**Figure 3:**
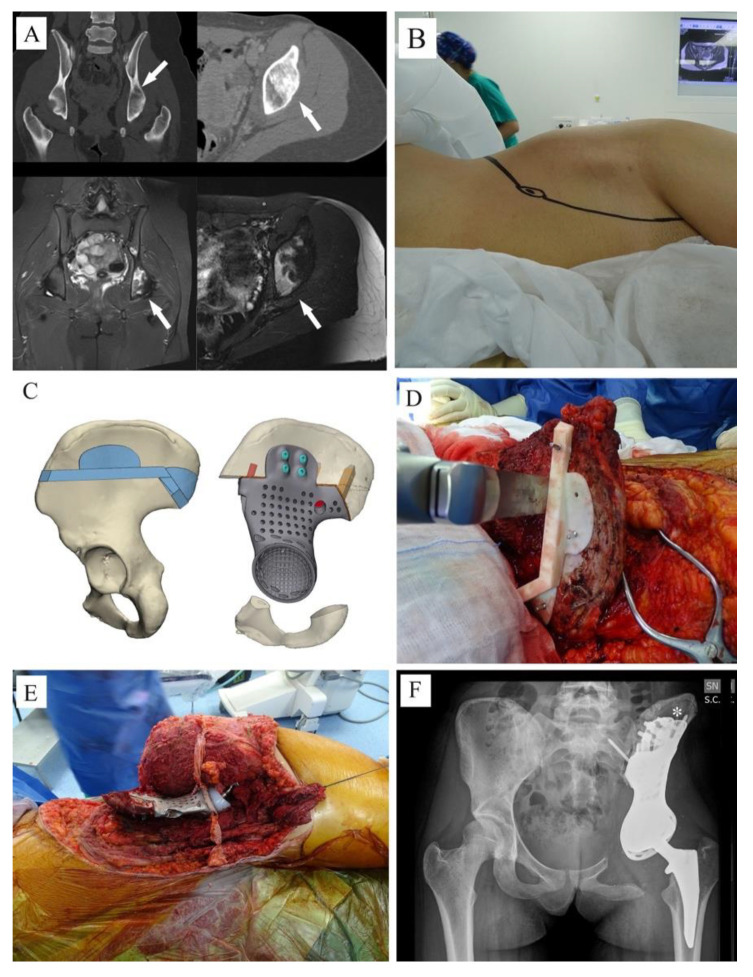
(**A**) CT-scan and MRI showing a Ewing sarcoma involving the acetabulum (white arrows); (**B**) due to ischiatic notch involvement, the young patient was positioned in lateral position and an ileo-inguinal approach was performed; (**C**,**D**) the tumor was removed using cutting guides; (**E**): an intraoperative picture showing the 3DPTCMP used to reconstruct the hemipelvis and a neck-sparing short press-fit stem prosthesis used to restore the proximal femur; (**F**) postoperative X-ray showing the “wafer fixation” (white asterisk); no connection to the residual symphysis or to the ischium was adopted.

When the tumor is located in the acetabular area, the incision is performed along the anterior part of the iliac crest, prolonged along the lateral portion of the inguinal ligament and then distally along the vascular bundle (Figure 2A); when the tumor also involves the symphysis, the incision proximally bends slightly, to cross the inguinal ligament more medially (Figure 2B). If the tumor involves the iliac wing, the hip and the symphysis, the incision starts at the iliac wing and is prolonged medially towards the umbilicus and then extended distally through the semilunaris line and, once crossed the inguinal ligament, is drawn along the anterior surface of the thigh (Figure 2C). When the tumor even involves the sacrum-iliac joint, the incision has to be extended posteriorly along the joint line with the patient in the lateral position (Figure 2D and Figure 3A,B).

When the tumor involves the ischium, it can be resected both from an anterior or a posterior approach, based on the case; the anterior approach is suggested when the Obturator Foramen is not involved in the tumor, otherwise the incision would have to be prolonged more distally to reach the ischium from behind. When necessary, the femoral head can be cut to better visualize the ischium.

The ileo-inguinal approach allows a good identification of the ileo-femoral neurovascular bundles; each component can be isolated, giving the advantage of a better visualization of the symphysis and of the internal pelvis, but it exposes the vessels and femoral nerve to possible damage, as happened in one case with rupture of the external iliac vein; actually, neurovascular bundles should be identified and protected, together with the iliopsoas muscle, whenever possible; if this is completed, the visibility is lower but the ileo-femoral vein ad artery and the femoral nerve are more protected from accidental damages.

Tumors were then removed using customized cutting guides to perform the osteotomies designed during the project (Figure 3C,D).

The margins were wide in 12 cases, marginal in one case and intralesional in one case of G2 Chondrosarcoma.

**Figure 4:**
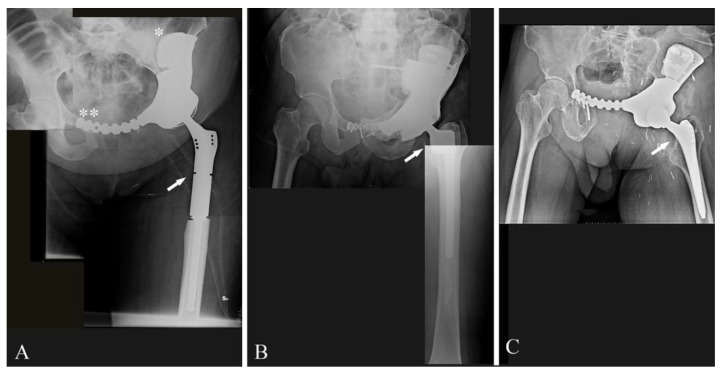
(**A**) Post-operative X-ray showing reconstruction after G1 chondrosarcoma of the left hemipelvis with a 3DPTCMP fixed to the residual iliac bone with a “wafer” system (white asterisk) and then to the residual symphysis with a plate (double white asterisk); proximal femur was reconstructed with a modular prosthesis and a constrained cup (white arrow) (case 1); (**B**) post-operative X-ray showing reconstruction after resection of a left hemipelvis G2 chondrosarcoma; reconstruction was performed with a short modular femoral prosthesis (white arrow) and a constrained cup cemented in a 3DPTCMP (case 5); (**C**) post-operative X-ray showing reconstruction after resection of a left hemipelvis G3-Chondrosarcoma; reconstruction was performed with a standard femoral stem prosthesis (white arrow) and a constrained cup cemented in a 3DPTCMP (case 12).

In the 12 cases in which resections involved the acetabulum, reconstructions were performed with a 3DPTCMP that reproduced the acetabulum wherein a cup component was cemented (Figure 3E,F). Of those 12 cases, seven cases underwent hip reconstruction with a constrained system and five cases with a dual mobility system. The femur was reconstructed with a 13 cm modular cemented stem prosthesis in one case (Figure 4A), with a 3 cm minimal resection modular cemented-stem prosthesis in four cases (Figure 4B), with a standard press-fit-stem prosthesis in four cases (Figure 4C) and with a neck sparing short press-fit-stem prosthesis in another case (Figure 3F).

Considering the 11 cases in which the symphysis was completely or partially resected, the connection with the residual symphysis failed in the first case (case 10) because of an insufficient osteotomy precision; the connection was made with an extension plate included in the prosthesis design in eight cases (Figure 4A). In the last two cases the prostheses did not present any connection to the residual symphysis (Figure 3F), but an alloplastic fascia lata was used to reconstruct the inguinal ligament to decrease the risk of inguinal herniation of viscera.

The ischium was fully resected in five out of 11 cases; in the remaining six cases in which a residual segment was present, it was connected to the prosthesis with a screw in three cases; no connection was made in the other three patients.

**Figure 5:**
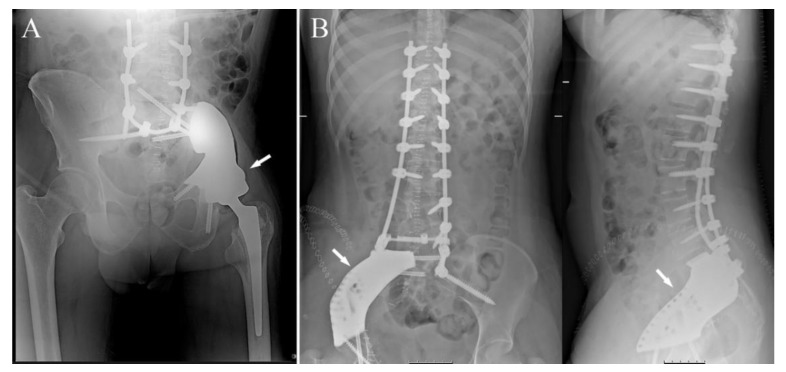
(**A**) post-operative X-ray showing reconstruction after resection of a Ewing sarcoma of the left hemipelvis with sacrum-iliac involvement; reconstruction was performed with a standard femoral stem prosthesis and a dual-mobility cup cemented in a 3DPTCMP (white arrow) linked to a spine stabilization to neutralize the cutting forces acting on the bone–prosthesis interface (case 7); (**B**) post-operative X-ray showing reconstruction after resection of a mesenchymal high-grade chondrosarcoma of the right sacrum-iliac joint; reconstruction was performed with a 3DPTCMP (white arrows) linked to a long spine stabilization (case 14). The two surgeries were performed by two different surgeons in the two different hospitals; the length of spine stabilizations was due to personal preferences.

In one out of the 12 acetabular cases, the resection also included the sacroiliac joint (case 7); the 3DPTCMP also reconstructed that area and was fixed with screws to the body of S1 (Figure 5A).

Moreover, the prosthesis was linked to a spine stabilization system to neutralize cutting forces on the sacrum-iliac Joint (Figure 5A).

In the last two cases, in which the tumor only involved the sacrum-iliac joint, sparing the acetabulum, the 3DPTCMPs restored the gap and were fixed with screws to the sacrum and to the residual ileum; in both cases, a spine stabilization system linked to the prosthesis was added to obtain higher stability (Figure 5B) (case 14).

In those cases, a supplementary posterior median incision was performed to allow spine stabilization and arthrodesis; in one case, stabilization was performed at the same time of tumor extirpation and pelvic reconstruction; in the remaining two cases, spine stabilization was performed in a different timing, after and before index surgery.

Nerve cut: in one case the sciatic nerve was cut because it was comprised in the tumor; the patient walks with two crutches, also because of excessive weight. In one case, the femoral nerve was cut with a resulting deficit of the quadriceps muscle.

The median operation time was approximately 10 h (average 542 min; median 600; range 180–780 min).

The median hospital stay was approximately 22 days (average 23.3, range 5–53 days).

### 3.5. Chemotherapy

Both patients affected by Ewing sarcoma and the patient affected by mesenchymal chondrosarcoma were treated with neoadjuvant chemotherapy; one patient, the only one with metastatic disease, affected by epithelioid hemangioendothelioma, underwent several courses of chemotherapy before and after surgery; two patients underwent chemotherapy after the operation for systemic disease progression; no chemotherapy was performed in the remaining cases. 

### 3.6. Radiotherapy

No cases were treated with radiotherapy.

### 3.7. Complications

Early Complications: Eight out of 14 patients experienced early complications (57.1%); the most frequent one was wound dehiscence, which occurred in five cases; three out of five were aseptic dehiscences, whereof two were resolved with revision; the last one died from secondary iliac vein rupture after revision, at two months of follow-up. The last two were infective dehiscences, resolved with multiple debridements. The other early complications were a deep venous thrombosis resolved with medical therapy, a peroneal nerve palsy and an unsatisfying reconstruction in the first case performed, in which the anterior plate of the prosthesis did not adapt to the symphysis, probably due to imperfect osteotomies (case 10).

Late complications: one patient, affected by Ewing sarcoma, underwent deep infection and following systemic sepsis, which was resolved with hemipelvis prosthesis and spine stabilization removal and positioning of an antibiotic cement spacer at two months from index surgery; after one year, the patient had a rib metastasis, treated firstly with adjuvant chemo- and radiotherapy and then with surgical extirpation (case 7). At present, after 12 months from the last surgery, the patient is apparently free of disease and is due to receive a spacer exchange with a new implant. Another patient, affected by low grade chondrosarcoma and who underwent multiple revisions for wound dehiscence and bleeding, had an iliac vein rupture at two months from surgery (case 5).

### 3.8. Local Recurrence

One out of 14 cases, affected by G3 Chondrosarcoma, experienced local recurrence at 36 months from index surgery, which probably arose in the biopsy track that had not been completely excised; it was treated with surgical extirpation of the nodule and chemotherapy for disease progression (case 3).

**Figure 6:**
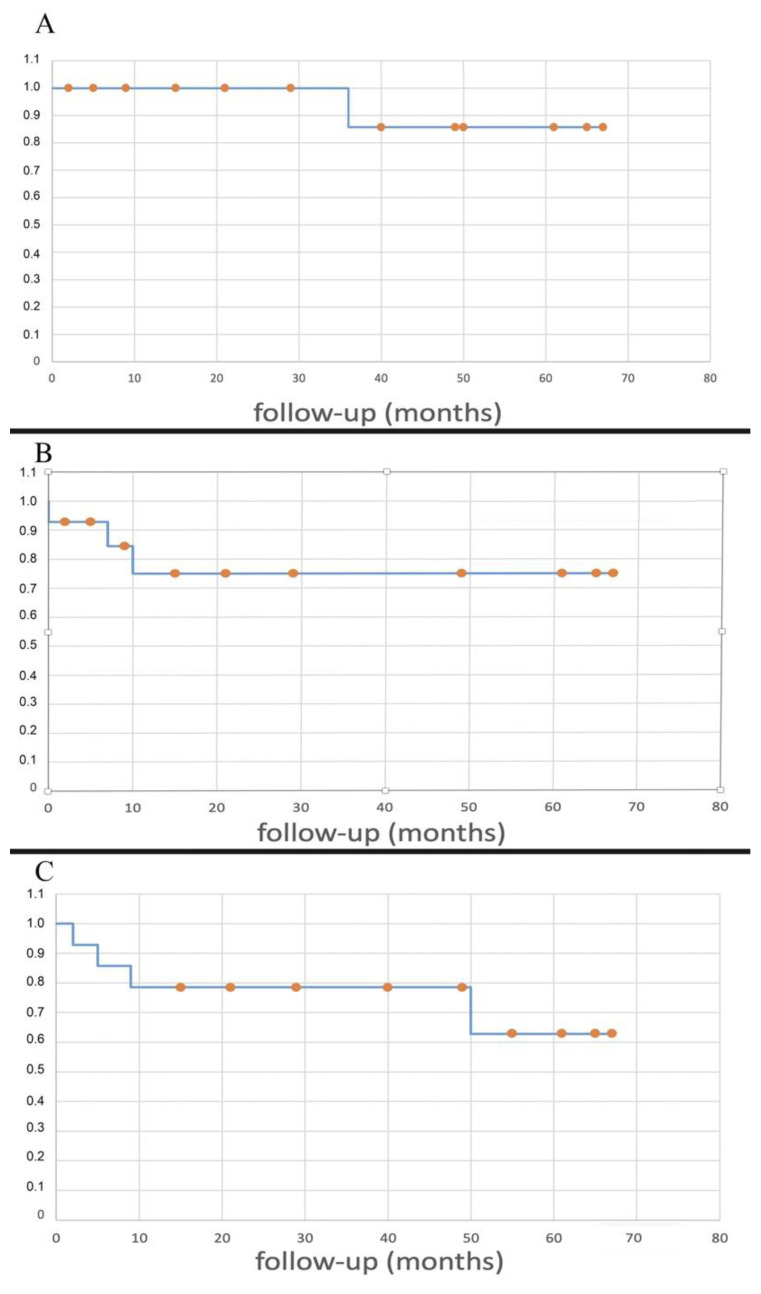
Kaplan–Meier curves showing LRFS (**A**), DMFS (**B**), OS (**C**).

The 5y-LRFS was 85.7%. The related Kaplan–Meier curve is shown in Figure 6A.

### 3.9. Metastases

One patient affected by hemangioendothelioma was already metastatic at index surgery (case 6); two cases had metastases at seven and 12 months of the lung and a rib, respectively, and both were surgically treated with resection (case 3 and 7). The first one underwent a first metastasis resection after diagnosis, successive further pulmonary progression and, at today, is on chemotherapy.

The second was firstly treated with radio- and chemotherapy and after about one year he underwent surgical rib extirpation.

At present, he is apparently free of disease and is due to have a new prosthetic reconstruction after the removal of the first one for infection. 

The 5y-DMFS was 75.0%. The related Kaplan–Meier curve is shown in Figure 6B.

### 3.10. Survival

The five-year overall survival was 62.9%. No deaths caused by the tumor were reported, except for a patient that was already metastatic at index surgery (case 6); the other three deaths occurred at two months from the tumor extirpation from iliac vein rupture (case 5), at 11 months from sepsis caused by an erysipelas (case 4), and at 12 months because of a heart attack (case 1). The Kaplan–Meyer curve related to the OS is shown in Figure 6C.

### 3.11. Functional Results

At present, just considering the living patients, at the average follow-up of 42 months (median 44.5, range 15–67, DS: 20.6), two patients walk normally without any sign of limping, one patient is able to walk without crutches with a slight limp, one patient is able to walk without crutches but with the affected limb 70° externally rotated, three patients need a crutch, two patients walk with two crutches and the last patient is due to have spacer removal and new reconstruction at 40 months from index surgery and 12 months from rib metastasis removal. The average MSTS score, valued on the living patients with 3DPTCMP, was 46.3% (min 16.7%–max 80%, median 43.3%).

At the latest follow-up, no signs of 3DPTCMP loosening are reported; no case of femoral prosthesis dislocation occurred both in the constrained cup and the dual-mobility cup groups. No cases of stem loosening were reported, both in the cemented and the uncemented groups. Two cases of loosening of the symphysis synthesis and related osteolysis were reported (case 3, 8); a case of ischium loosening also occurred with screw rupture (case 8).

## 4. Discussion

Reconstruction after resection of pelvic tumors is a challenging issue in oncological orthopedics. Modular prostheses are able to assure length and restore joints in the limbs; this is possible because both upper and lower limbs have a simple anatomy and the length of the segment is the only parameter that must be evaluated [18].

Conversely, anatomy is extremely complex in the pelvis where the other diameters also have to be taken into account. To our knowledge, modular prostheses for pelvic reconstruction are commercially available, but their use is quite limited [19,20].

Therefore, reconstruction is usually performed using massive composite allografts, composed by an alloplastic hemipelvis segment which is cut intraoperatively to adapt to the gap and stabilized by a plate and screws plus a standard total hip prosthesis [21].

This technique presents several weaknesses; first of all, the availability of allograft hemipelvises is limited; moreover, it is difficult to find an allograft with anatomic characteristics similar to those of the resected segment. The second factor is related to the mechanical resistance; indeed, osteointegration is limited to the area of contact between the allograft and patient’s bone for less than 2 cm; the major part of the allograft remains inert and is progressively reabsorbed, causing mechanical collapse [21,22,23].

The application of additive techniques allows us to produce custom-made prostheses with more affordable costs and assuring a more effective and more durable reconstruction

### 4.1. Indications

Indications for reconstruction with 3DPTCMP after resection of pelvic tumors has to be valued considering several factors:

- The patient’s age: 3DPTCMPs could be unsuitable in young ages because the pelvis still has to reach its ultimate size, whereas reconstruction in very old patients could give fixation and mobilization problems due to low bone quality.
- The diagnosis: the time needed to produce the 3DPTCMP has to be taken into account. At present, fabrication requires an average of four weeks, so patients affected by low-grade malignant tumors are more suitable than those with high-grade tumors. Obviously, the prosthesis can be produced during neoadjuvant chemotherapy in the case of high-grade tumors. Indeed, chondrosarcoma is the main histology treated, both because it is the most frequent malignant bone tumor in the pelvis and because its grade of aggressiveness is usually compatible with prosthesis making. In the present series, two cases of Ewing’s sarcoma also underwent reconstruction with 3DPTCMP. In these cases, chemotherapy was entirely performed in a neoadjuvant setting, considering that possible surgical infection could have played against chemotherapy prosecution. Indeed, one of the two cases had sepsis caused by urinary catheter infection; this caused a hardware contamination and removal was necessary to resolve the septic shock. The patient is due to have cemented spacer removal and new reconstruction, 12 months after rib metastasis extirpation and 40 months from index surgery (case 7).
- The site: the aim of reconstruction after pelvic resection is to restore hip stability by giving the articulation a pivot. Acetabulum sparing is the keystone. Reconstruction can be performed with a hip prosthesis that includes a cup with an iliac stem when part of the anterior or posterior column is spared. When resection includes the acetabular roof (Figure 3A), an acetabulum with an iliac stem does not have sufficient bone support, so 3DPTCMP is a possible solution (Figure 3C right side); the 3DPTCMP should be linked to a spine stabilization to neutralize vertical cutting forces when resection includes the sacrum-iliac joint (Figure 5A,B).

### 4.2. Reconstruction

**Figure 7:**
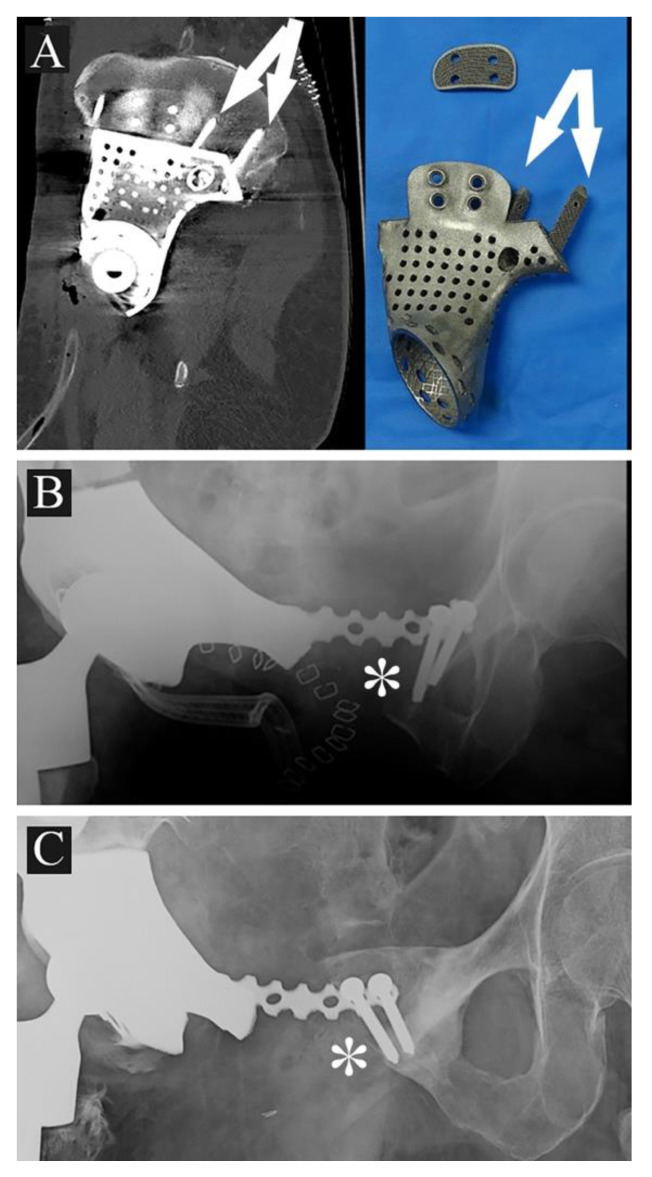
(**A**) 3DPTCMP with double iliac stem (white arrows) (case 9); (**B**) 3DPTCMP with connection to residual symphysis (white asterisk); (**C**) the same connection showed in (**B**) displaying mobilization and osteolysis (white asterisk).

Several problems are still debated in literature regarding the 3DPTCMP:

- Prosthesis shape: most prostheses were constructed trying to reproduce normal bone anatomy (Figure 4A–C). Actually, this tactic could cause problems as wound dehiscence due to skin decubitus on the prosthesis; taking this into consideration, the option of designing a low-profile prosthesis has to be taken into account (Figure 5A,B): when the tumor involves the iliac wing, the prosthesis could simply connect the sacrum-iliac joint to the hip without reproducing the iliac wing’s shape (Figure 5A,B). Currently, several prosthesis designs are proposed in the literature.
- The fixation: several solutions are possible; the first prostheses had a voluminous plate as an extension, which was fixed with screws to another independent plate on the other side of the iliac wing (“wafer fixation”) (Figure 4C); this technique could cause necrosis of the bone included between the plates, so, at present, an iliac stem is preferred to obtain primary stability, decreasing the size of the wafer fixation. In the last cases of the present series, the prostheses were produced with one or two stems which were inserted press-fit in the residual wing, obtaining a good primary stability with a probable lower damage of the bone’s vascular supply and a better osteointegration (Figure 7A). Park et al. in 2019, using a finite element analysis, showed how the shape of the plates of fixation play an important role in stress distribution. Further studies are advocated to find the best solutions [24].
- The pelvic ring restoration: several authors sustain the importance of restoring the connection between the sacrum-iliac area, the acetabulum and the symphysis [25,26]; other authors sustain a non-anatomical reconstruction [26,27]. Connection to the symphysis could be a more stable fixation at first (Figure 7B), but in the long term, this technique could increase the risk of osteolysis in the symphysis (Figure 7C) (case 3) and prosthesis loosening. Indeed, a connection with the symphysis can transfer the stress forces to the bone–prosthesis interface at the acetabular level every time that weight-bearing is on the contralateral side; contrariwise, when weight-bearing is on the side of the prosthesis, the stress forces are transmitted to the prosthesis–bone symphysis interface with hypothetical easier mobilization. Based on these concepts, connection to the symphysis has to be well evaluated; actually, a fascia lata allograft can be used to reconstruct the inguinal ligament and support the abdominal viscera when necessary (Figure 7C). The fascia lata also decreases the friction between the vessels and the prosthesis and therefore the risk of their damage, as happened in one case of our series, in which the patient died from iliac vein rupture (case 5). Instead, reconstruction of the ileo-pubic ramus has an important biomechanical value; indeed, it represents a fulcrum with the iliopsoas muscle, allowing it to act as a hip flexor; when the ileo-pubic ramus is not reconstructed, the iliopsoas collapses inside the pelvis, acting more as an adductor muscle, with a consequent loss of flexion activity. Based on these considerations, while symphysis fixation presents a rationale, ischium fixation is not recommended because it would transmit cutting forces to the bone–prosthesis interface every time the patient sits down.
  When the tumor also involves the Enneking and Dunham T1 and 4 areas, spine stabilization is suggested to neutralize the cutting forces that act on the prosthesis (Figure 4A,B); in these cases, a median incision is completed to perform a bilateral stabilization; bone fusion is suggested to decrease the risk of pedicle screw loosening. This surgical step can be performed during tumor removal surgery or in a second moment to decrease the impact of a massive surgery on the patient. Actually, spine stabilization could become unnecessary in the future if osteointegration improves. A possible solution to improve osteointegration could be the use of a composite prosthesis composed by a 3DPTCMP as a scaffold for a vascularized autologous bone graft as a fibular flap; in that case, the 3DPTCMP should assure primary stability, whereas the fibular flap should give osteointegration and stability without the need of spine stabilization. Recently, Lu et al. proposed a similar composite structure for diaphyseal reconstruction after bone tumor removal with good results; actually, that technique could also be suggested for reconstruction in the Enneking and Dunham T 1 and 4 areas when the acetabular roof is spared [28].
- Hip reconstruction: a prosthetic cup is cemented inside the prosthetic acetabulum. In the first cases, a constrained system was used to reduce the risk of femoral prosthesis dislocation (Figure 4A,B); later on, a dual mobility system was adopted. Indeed, constrained hip prostheses transfer traction stress forces caused by the weight of the inferior limb directly on the bone–prosthesis interface while the dual mobility system is a less rigid strategy which should guarantee a better cushioning and may be at lower risk of prosthesis failure (Figure 3E,F). Both strategies are probably effective in preventing femoral head dislocation.

### 4.3. Complications

As reported in the literature, wound dehiscence and infections are the main complications; they are likely due to several concomitant factors such as the length of the incision, the duration of the surgeries and the thickness of the soft tissues covering the prosthesis; moreover, the residual dead space after resection and the related seroma can easily be contaminated by bacteria during surgery or afterwards.

A possible strategy to reduce the infection rate could be the use of a myocutaneous flap; the one most often used in literature is the vertical rectus abdominis myocutaneous flap, harvested from the ipsilateral or from the contralateral side [29,30]. Alternatively, the pedicle anterolateral flap can also be considered [31].

Plastic flaps present a double advantage: they decrease the dead space and the consequent seroma, reducing the risk of bacterial colonization; moreover, they decrease the tension on the wound closure and, consequently, of secondary wound failure.

Another risk factor for infection derives from the duration of the surgeries and the long exposures that can cause bacterial contamination of the prosthesis. The possibility to cover 3DPTCMPs with bioactive materials such as silver ions, already performed in modular prostheses, could decrease infection rate. 

Another possibility that was adopted in the present series is the application of a calcium matrix as a carrier for antibiotics with controlled release; it is also possible to provide the prosthesis with small niches to accommodate it (Figure 3C,E and Figure 7A) [32].

Additionally, the application of an antibacterial hydrogel coating to the prosthesis’ surface was demonstrated to be an effective strategy to decrease biofilm formation, reducing infections and antibiotic resistance [33].

Actually, in high-risk cases, the possibility to perform resection and a temporary reconstruction with a spacer in a first surgical step and then reconstruction at a second time could be considered.

When the incision is near the pelvis, a temporary colostomy could be a valid possibility to decrease the risk of intestinal bacteria contamination.

In the presented series, a case of iliac vein rupture was reported; the causes could be associated both to revision surgeries and to a direct damage from the rough prosthetic surface. To decrease the risk, we suggest isolating the iliofemoral neurovascular bundles together, lifting them from the ileo-pubic ramus; this protects the vessels from possible damage by the rough prosthetic surface and the femoral nerve from possible sprain; otherwise, a homoplastic fascia lata can be used both to protect vessels from the prosthesis surface and reconstruct the inguinal ligament, giving contention to abdominal viscera

### 4.4. Cost Analysis

The average cost of a pelvic prosthesis was 12000 EUR plus 3000–6000 EUR in the case of spine stabilization based on its length; the cost of the hip prosthesis was not considered because it was included in the case of allograft composite reconstruction as well; the cost for a hemipelvis allograft is between 4500 EUR and 6000 EUR, depending on the weight, plus the price of plates and screws necessary for stabilization. The 3DPTCMP is more expensive but with the wide-spreading of the technology, the price should decrease consistently. 

This study presents some limitations; first of all, the retrospective design and related problems due to the consideration of data from two different centers; secondly, the small sample size due to the rarity of the disease and the indications.

Nevertheless, it allows us to identify some hot topics and questions which have to be clarified and discussed in more extensive series.

## 5. Conclusions

The 3D-printed titanium custom-made prosthesis is an effective resource for reconstruction after resection of tumors located in the pelvis. Some topics need to be clarified, such as the best fixation to the iliac wing and to the symphysis; effective strategies have to be identified to decrease the risk of infection. Further studies are necessary to value long-term results.

## Figures and Tables

**Table 1:** Patients’ main characteristics.

N°	PT	Age	Dia	G	Enn C	D&E C	SITE	M	3DP	Loos	SS	Loos	Cup	Disl	Stem	Type	Loos	Sym Conn	Loos	Is Conn	Loos	FU	R	M	Status
1	MS,F	58	CS	G1	IB	T2-T3	pf, ace	w	yes	no	no	.	con	no	cem	13 cm-mp	no	yes	no	res	.	65	no	no	NED
2	FA,F	74	CS	G2	IB	T1-T4	SIJ	w	yes	no	yes	no	.	.	.	.	.	.	.	.	.	12	no	no	d (heart attack with NED)
3	CC,M	53	CS	G3	IIB	T2-T3	ace, is, il-p-r	w	yes	no	no	.	con	no	cem	minimal mp	no	yes	yes	res	.	55	36	7m (lung)	alive with lung metastasis
4	BM,M	65	CS	G3	IIB	T1-T2	ace	w	yes	no	no	.	con	no	cem	minimal mp	no	yes	no	yes	no	11	no	no	d (Erisipela with NED
5	CG,M	72	CS	G2	IB	T2-T3	ace, il-p-r	w	yes	no	no	.	con	no	cem	minimal mp	.	yes	no	failed	.	2	no	no	d (iliac vein rupture)
6	LA,F	57	MEH	LG	III	T2-T3	ace, il-p-r	w	yes	no	no	.	con	no	cem	minimal mp	no	yes	no	yes	no	50	no	already present	d (for lung progressionw/o local disease
7	PP,M	48	ES	HG	IIB	T1-T2-T4	ace, SIJ	w	yes	r	yes	r	dm	no	cem	standard	.	.	.	.	.	40	no	12 (rib)	NED He is due for temporary spacer exchange with new prosthesis
8	LA,F	50	CS	G3	IB	T2-T3	ace, il-p-r	w	yes	no	no	.	dm	no	pf	standard	no	yes	yes	yes	yes	15	no	no	NED
9	BG,F	15	ES	HG	IIB	T1-T2-T3	ace, Il, is, Il-p-r	w	yes	no	no	.	dm	no	pf	mini	no	no	.	no	.	21	no	no	NED
10	IM,M	38	CS	G3	IIA	T2-T3	ace, il-p-r	m	yes	no	no	.	con	no	pf	standard	no	failed		res	.	67	no	no	NED
11	CAM,F	53	CS	G2	IIB	T2-T3	ace, il-p-r, is	i	yes	no	no	.	dm	no	pf	standard	no	yes	no	res	.	49	no	no	NED
12	FL,M	68	CS	G3	IIB	T2-T3	ace, il-p-r	w	yes	no	no	.	con	no	pf	standard	no	yes	no	res	.	61	no	no	NED
13	CP,F	58	CS	G2	IIA	T2-T3	ace, is	w	yes	no	no	.	dm	no	pf	standard	no	no		no		29	no	no	NED
14	KK,F	20	CS	MHG	IIB	T1-T4	il, SIJ	w	yes	no	yes	no	.	.	.	.	.	.		.	.	15	no	no	NED

PT: patient, F: female, M: male, Dia: diagnosis, CS: chondrosarcoma, MEH: Metastatic Epithelioid Hemangioendothelioma, ES: Ewing’s sarcoma, G: grade, LG: low-grade, HG: high-grade, MHG: Mesenchimal high-grade, Enn C: Enneking classification, D&E C: Dunham and Enneking classification, pf: proximal femur, SIJ: sacrum-iliac joint, ace: acetabulum, is: ischium, il-p-r: ileo-pubic ramus, il: ileum, Sym: symphysis, M: margin, w: wide, m: marginal, i: intralesional, 3DPTCMP: 3D-printend titanium custom-made prosthesis, loos: loosening, r: removed, SS: spine stabilization, disl: dislocation, conn: connection, Con: constrained, dm: dual mobility, pf: press-fit, cem: cemented, mp: modular prosthesis, res: resected. FU: follow-up (months), R: recurrence (months), M: metastasis (months), d: dead (months).

**Table 2:** Main epidemiology characteristics, histologies, surgery and related complications.

Epidemiology	14 Patients (6 Males, 8 Females)
Histologies	- Chondrosarcoma: 11 cases (G3:5, G2:4, peripheral G1:1, mesenchymal CS: 1) - Ewing’s Sarcoma: 2 - Epithelioid hemangioendothelioma: 1
Enneking and Dunham classification	- T2-T3: 9 - T1-T2-T4: 3 - T1-T4: 2
Enneking staging	IB: 4, IIA: 2, IIB: 7; III: 1
Symptoms	- Pain: 13 out of 14 - Neurological Symptoms: 6 (sciatic: 4, crural: 1, neurological bladder: 1) - Hip anchyloses: 1
Surgeries	- Acetabulum reconstruction: 12 (SI joint included with spine stabilization: 1); - SI joint with spine stabilization: 2
Margin	Wide: 12, Marginal: 1, Intralesional: 1
Early complications:	8 patients - wound dehiscence: 5 (aseptic: 3, septic: 2) - deep venous thrombosis: 1 - peroneal nerve palsy: 1 - unsatisfactory reconstruction: 1
Late complications	- Deep infection and sepsis: 1 - Massive bleeding: 1 (vein rupture)
Local recurrence	1 case
Distant Metastasis	3 (one already metastatic at index surgery)

## Data Availability

Data available on request due to restrictions, e.g., privacy or ethical.
